# Sitting Less, Recovering Faster: Investigating the Relationship between Daily Sitting Time and Muscle Recovery following Intense Exercise: A Pilot Study

**DOI:** 10.3390/jfmk9010024

**Published:** 2024-01-29

**Authors:** Jaime Rodden, Dolores G. Ortega, Pablo B. Costa

**Affiliations:** Exercise Physiology Laboratory, Department of Kinesiology, California State University, Fullerton, CA 92831, USA; jaimerodden@csu.fullerton.edu (J.R.); dortega6@huskers.unl.edu (D.G.O.)

**Keywords:** prolonged sitting, resistance exercise, interrupted sitting, C-reactive protein, creatine kinase, myoglobin, white blood cells, inflammatory markers

## Abstract

(1) There is growing concern surrounding the adverse effects of prolonged sitting on health, yet its impact on post-exercise recovery remains relatively unexplored. This study aimed to better understand the potential influence of habitual prolonged sitting on recovery time and the unfavorable impact prolonged sitting may have on time to recovery, as assessed by muscle damage and inflammatory markers and an isokinetic dynamometer. (2) Nine college-age men (mean age ± *SD* = 22.1 ± 3.1 years, body mass = 80.9 ± 15.7 kg, height = 171 ± 9.0 cm, Body Mass Index (BMI) = 27.6 ± 4.9 kg·m^2^) participated in an exhaustive exercise protocol. Creatine Kinase (CK), Myoglobin (Mb), C-Reactive Protein (CRP), White Blood Cell Count (WBC), Peak Torque (PT), and muscle soreness were measured at baseline and 0, 24, 48, and 72 h post-exercise. Dietary and exercise logs were maintained during the 5-day testing procedure. (3) No significant differences were observed in muscle damage markers (CK [*p* = 0.068] and Mb [*p* = 0.128]), inflammatory markers (CRP [*p* = 0.814] and WBC [*p* = 0.140]), or PT [*p* = 0.255]) at any time point. However, a significant positive correlation was found between daily sitting time and the percent increase in CK concentration from 0 h to 72 h (*r* = 0.738, *p* = 0.023). Strong correlations were also noted between prolonged sitting and percent change in Mb concentration at 48 h (*r* = 0.71, *p* = 0.033) and 72 h (*r* = 0.889, *p* = 0.001). There was a significant two-way interaction for time × velocity (*p* = 0.043) for PT with a simple main effect for time at 60°·$s^{-1}$ (*p* = 0.038). No significant associations were detected between daily carbohydrate or protein intake and recovery markers (*p* > 0.05). (4) The findings suggest minimizing daily sitting time may expedite and potentially aid muscle recovery after an intense exercise bout, although further research is warranted to validate these findings.

## 1. Introduction

The benefits of physical activity are well-known [1,2,3,4,5], but the adverse effects of prolonged sitting are a relatively new topic of interest that has yet to be studied extensively. Prolonged sitting encompasses time spent inactive, sitting, or reclined while participating in sedentary behaviors with energy expenditure ≤ 1.5 METs (metabolic equivalent of task) [6]. Although prolonged sitting has yet to be defined conclusively, a “prolonged bout” is typically reported as ≥30 min of uninterrupted sitting [7,8,9,10], with some studies reporting sitting bouts as >20 min [11,12]. As has been previously reported, prolonged sitting is independently associated with various adverse health consequences, regardless of physical activity level [13]. These health outcomes include, but are not limited to, cardiovascular disease mortality, cancer mortality, cancer incidence, and diabetes [14,15,16,17]. With the rise in technological advancements, societal changes, and the shift towards remote jobs, prolonged sitting is increasing, currently estimated to be 9–10 h per day for the average adult in developed nations [18]. Furthermore, total daily energy expenditure is declining [19,20]. Recent investigations have examined the effects of both reducing and increasing prolonged sitting time and concluded that prolonged sitting is associated with adverse health risks independent of daily physical activity level [13,21].

The World Health Organization (WHO) has issued physical activity recommendations of 30 min of moderate-intensity exercise for five days per week and two or more days of muscle-strengthening exercises [22]. However, only 24.2% of adults [23] consistently meet the WHO physical activity recommendations. This has led to a considerable body of literature focused on exploring the adverse implications of habitual prolonged sitting and a lack of physical activity [13,17,24,25,26,27,28]. The predominant physiological markers investigated thus far indicative of the negative impacts of prolonged sitting include observational and experimental studies that have identified acute and chronic effects concerning high plasma glucose [9], endothelial dysfunction [29], high levels of low-density lipoprotein (LDL) and total cholesterol [18,30], suppression of high-density lipoprotein (HDL) [31,32], elevated plasma triglycerides, decreased insulin sensitivity [33], heightened C-reactive protein (CRP) [34,35,36,37], fibrinogen [38,39,40], and white blood cell (WBC) count [39,41].

The physiological markers creatine kinase (CK) and myoglobin (Mb) are often used to assess muscle damage [42,43,44]. For instance, Gordon III et al. [42] and Cockburn et al. [43] demonstrated significant increases in Mb and CK compared to baseline in the subsequent days following a strenuous exercise bout. Likewise, Crosier et al. [45] reported similar findings, with CK and Mb concentrations significantly elevated 24 and 48 h after a strenuous quadriceps exercise protocol. Although the potential association between habitual prolonged sitting and heightened muscle damage following strenuous exercise has yet to be investigated, it can be speculated that there is an association [46,47,48]. For instance, past studies have shown elevated CK levels in untrained and sedentary individuals compared to trained persons or athletes following an exercise bout [49,50,51,52]. Vincent and Vincent [49] found CK concentrations were significantly higher in untrained individuals following a strenuous weight-training regimen compared to trained weightlifters. Similar results were evident in a study by Fehrenbach et al. [52], who compared trained endurance athletes to sedentary controls. In an investigation by Garry and McShane [51], trained football players demonstrated significantly lower CK concentrations following strenuous exercise compared to untrained individuals. To date, research on muscle damage has focused predominantly on athletic performance and comparisons between trained and undrained subjects [53,54]. To our knowledge, research on muscle damage marker responses to exercise in those who demonstrate heightened habitual prolonged sitting is nonexistent.

The concept of prolonged sitting is still relatively novel, but research is rapidly growing [7,8,9,10,11,13,17,18,24,25,26,27,28]. The initial research on prolonged sitting predominantly includes the impact of interrupting prolonged sitting by various durations, intensities, and types of exercise on metabolic markers, the metabolic implications of chronic prolonged sitting, and prospective cohort studies [11,18,21,31,55,56,57,58,59]. Still, a more detailed evaluation of the literature reveals several gaps and shortcomings. Accordingly, the literature on the effects of prolonged sitting and lack of adequate physical activity on systemic inflammatory (CRP and WBC) [17,41,60,61] and muscle damage (CK and Mb) [62,63,64,65] markers is lacking. Furthermore, most existing literature has focused on acute inflammation responses to exercise, inflammation responses in older adults, overweight or obese adults, and long-term prospective studies [41,60,66,67,68]. Considering this, a population that has yet to be investigated in sufficient detail is college-age adults [69,70,71]. A significant decline in regular physical activity is observed in college students, highlighting the importance of examining this population [72,73,74]. Clinically, a better understanding of the impact prolonged sitting time and lack of physical activity have on inflammation and muscle damage in apparently healthy college-aged individuals could provide additional predictive measures for enhanced detection of cardiovascular disease (CVD) risk and performance capacity [69]. In addition, determining the impact prolonged sitting and inactivity have on inflammatory and muscle damage markers in college students is crucial for providing college students and the public with feasible interrupted sitting guidelines. In a culture where daily sedentary time is rising [12,75], additional investigation into the relationship between prolonged sitting and inflammatory and muscle damage markers is imperative. 

To our knowledge, the influence of habitual, prolonged sitting on recovery as measured by muscle damage markers following strenuous exercise has yet to be explored. Recovery is often defined as the return to baseline performance measures or by a reduction in muscle damage (CK and MB) and systemic inflammatory (WBC and CRP) marker concentrations in response to exercise [42]. A better understanding of the unfavorable impact prolonged sitting may have on recovery is of paramount importance. This heightened understanding is crucial since extended time to recovery can decrease exercise tolerance, therefore likely affecting adherence to an exercise training program [44,76] and difficulty in upholding the WHO physical activity recommendations [22]. Recent literature suggests that breaking up prolonged sitting time with light physical activity has the potential to minimize systemic inflammation [17,27,29]. However, there is an absence of research examining the impact of prolonged sitting on recovery. Therefore, the purpose of this study was to investigate the relationship between daily prolonged sitting time and time to recovery, as assessed by systemic inflammatory (CRP and WBC) and muscle damage (CK and Mb) markers following an exhaustive exercise bout. A secondary aim of this study was to explore whether recovery time was affected by daily physical activity level, daily carbohydrate intake, or daily protein intake. 

## 2. Materials and Methods

### 2.1. Subjects

Nine college-age men who met the inclusion criteria volunteered to participate in this study (mean age ± *SD* = 22.1 ± 3.1 years, body mass = 80.9 ± 15.7 kg, height = 171 ± 9.0 cm, BMI = 27.6 ± 4.9 kg·m^2^). All subjects were apparently healthy and recreationally active, as defined by the American College of Sports Medicine [77] and a health status questionnaire [78]. On average, subjects reported partaking in exercise for 6.8 ± 2.4 h per week [79]. Participants were free of lower-extremity injuries in the six months leading up to the study, as determined by a health and activity questionnaire [80]. Participants were asked to cease lower body exercise [81] and refrain from possible muscle soreness-reducing treatments such as massage, foam rolling, ice therapy, or topical analgesics throughout the duration of this study. Additionally, consumption of numerous substances was avoided for the duration of this study, including supplemental protein or branch chain amino acids in quantities > 2 servings/week, antioxidants or anti-inflammatory drugs, grapefruit and grapefruit juice, steroids, caffeine, HMB, and creatine [81,82]. Other exclusion criteria screened by the health status questionnaire included the use of any medications that could affect the outcome of the study [42] or a history of medical events such as cardiovascular disease, metabolic, renal, hepatic, or musculoskeletal disorders [82]. This study was approved by the university’s Institutional Review Board for Human Subjects. 

### 2.2. Research Design

**Figure 1:**
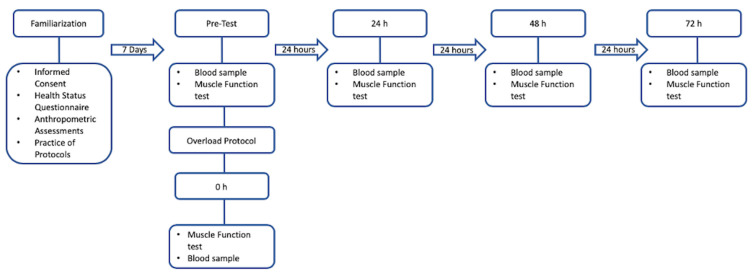
Research design.

The quasi-experimental study consisted of five separate visits to the laboratory (Figure 1). The initial visit was a familiarization day (visit 0), where the informed consent was read and signed, a Pre-Exercise Testing Health and Exercise Status Questionnaire was completed, and anthropometrics were recorded. Subjects became familiar with the testing protocols, including the isokinetic muscle function test, blood draw and overload protocols, and lifestyle logging application. After the familiarization visit, subjects returned to the lab for visit 1 of four a week later. Visit 1 included the pre-test, where participants completed their first blood draw, and the muscle function test. Testing consisted of isokinetic muscle peak torque using three concentric/eccentric knee extension repetitions at velocities of 60°·$s^{-1}$ vs. 180°·$s^{-1}$ vs. 300°·$s^{-1}$. After the pre-test, the overload protocol was performed, consisting of 8 sets of 10 concentric/eccentric knee extension repetitions at 60°·$s^{-1}.$ The overload protocol was immediately followed by the post-test (0 h test), and the remaining three follow-up sessions took place at 24, 48, and 72 h after the overload protocol.

### 2.3. Procedure

Subjects participated in an overload exercise protocol designed to induce muscle damage and muscle soreness to assess the impact of both peak torque and the muscle damage and inflammatory markers CK, Mb, CRP, and WBC. 

During the familiarization visit, height was measured to the nearest 0.1 cm using a stadiometer (SECA stadiometer, Chino, CA, USA), and body mass was measured using a digital scale (Ohaus ES Series scale, Parsippany, NJ, USA). Body mass index was calculated by dividing the subject’s body mass in kilograms by height squared in meters. Subjects were familiarized with the isokinetic dynamometer (Humac Norm CSMi, Stoughton, MA, USA), the muscle function test, and the overload protocol. Seated in the dynamometer chair, measurements for height, front and lateral position, lever arm length, seat front and lateral position, and knee axis of rotation were recorded to ensure replication for all testing sessions. At this time, subjects became familiar with the lifestyle MyFitnessPal web-based database (MFP) logging application. Lifestyle logging of food consumption, exercise, and prolonged sitting time (≥30 min uninterrupted) transpired for five days, starting 24 h before visit 1 and concluding with the completion of visit 4. 

The familiarization session was followed by visit 1, which occurred one week later. Visit 1 involved three separate segments: the pre-test, overload protocol, and post-0 h test. The pre-test began with a blood draw to establish baseline levels of muscle damage markers (CK and Mb) and inflammatory markers (CRP and WBC). Following the blood draw, the muscle function test was performed via the isokinetic dynamometer on the randomly assigned quadriceps to measure peak torque and soreness status. After the pre-test, the participant remained on the isokinetic dynamometer to perform the overload protocol. Immediately following the overload protocol, post-0 h testing concluded the visit, involving the same muscle function test and blood draw protocols. Following visit 1, participants returned the following 24, 48, and 72 h for visits 2–4, at which time the blood draw and muscle function test protocols were replicated. Each visit took place at approximately the same time of day (i.e., morning, afternoon, or evening).

### 2.4. Muscle Function Test Protocol

A dynamometer (Humac Norm CSMi, Stoughton, MA, USA) was used to assess isokinetic peak torque at the three different velocities of 60${}^{\circ}\cdot s^{-1}$, 180°·$s^{-1}$, and 300°·$s^{-1}$. Subjects were seated on the dynamometer with the randomly assigned leg secured in the machine, strapped at the shin, shoulders, and across the lap for optimal isolation of the limb strapped to be tested. The contralateral leg was placed behind the stabilization bar, and subjects were held onto the seat handles. The dynamometer was set in such a manner that the axis of rotation of the dynamometer aligned with the lateral femoral epicondyle. Prior to each testing velocity, a warm-up was conducted consisting of four knee extensions of increasing intensity at approximately 25%, 50%, 75%, and 100% of the subject’s perceived maximal output [80,83,84]. Each warm-up was followed by a 1 min rest [80,83,84]. The subject then performed three maximal repetitions, and the highest value of each velocity was recorded. Subjects were given verbal cues such as “kick”, “push”, and “resist” while completing each repetition [80,84].

### 2.5. Overload Protocol 

An adapted overload protocol previously demonstrated to induce extensive muscle damage was used to elicit blood marker responses [42,43,82,85]. The overload protocol consisted of a series of concentric and eccentric muscle actions of the quadriceps on the isokinetic dynamometer. Using the formerly tested limb, subjects completed a warmup set of 10 repetitions at 50% effort. Upon completing the warmup, the subject was asked to perform 8 sets of 10 repetitions of maximal quadriceps concentric and eccentric muscle actions on the dynamometer (Humac Norm CSMi, Stoughton, MA, USA) at 60°·$s^{-1}$ with one minute of rest between sets [82]. The range of motion was set between full knee extension (0°) and knee flexion (90°). Starting with flexion, subjects were asked to push against the dynamometer-level arm, moving their knee into full extension and back into flexion for each repetition. To ensure maximal effort throughout each extension, verbal encouragement was given during each repetition throughout the duration of each set. 

### 2.6. Lifestyle Logging Protocol

Subjects were asked to keep a log of all food and drink consumption, exercise, and prolonged sitting time using the application and web-based database MyFitnessPal. The lifestyle logging began 24 h prior to the pre-test and concluded at the time of visit 4, for a total of 5 days. In addition, subjects were asked to log any sitting time (commuting, seated social activities, time on social media, TV viewing, seated class time, etc.) lasting ≥ 30 min during all waking hours. 

### 2.7. Blood Sample Protocol

Blood samples were conducted by the campus Student Health Center using a standard venipuncture technique conducted by a trained phlebotomist. Upon arrival at the laboratory for each visit, beginning with the pre-test during visit 1, subjects were escorted over to the medical facility for the blood draw. Serum concentrations of CK, Mb, CRP, and WBC were measured from blood samples taken from the medial cubital vein of the preferred arm. The samples were collected into serum separator tubes under sterile conditions. All biochemical assays were run as per the manufacturer’s instructions. Once completed, subjects were escorted back to the laboratory for the remaining testing. The protocol was repeated upon completion of the post-0 h test, as well as at the beginning of visits 2, 3, and 4. 

### 2.8. Statistical Analysis 

Means and standard deviations for all variables were calculated. Correlational analyses comparing percent differences between 0 h and 24 h, 0 h and 48 h, and 0 h and 72 h were conducted to report the associations between lifestyle logging metrics (average daily prolonged sitting time, weekly physical activity, and daily protein and carbohydrate intake), inflammatory markers (WBC and CRP), and muscle damage markers (Mb and CK). Correlations were categorized as weak (0.10–0.39), moderate (0.40–0.69), strong (0.70–0.89), and very strong (0.90–1.00) [86]. A two-way repeated measures ANOVA was performed to compare the effect of time (pre vs. 0 h post vs. 24 h post vs. 48 h post vs. 72 h post) × velocity (60°·$s^{-1}$ vs. 180°·$s^{-1}$ vs. 300°·$s^{-1}$) on peak torque. Single factor (time) repeated measures ANOVAs were used to analyze changes in inflammatory (WBC and CRP) and muscle damage (CK and Mb) markers over time. All analyses were conducted using JASP (JASP Team version 0.16, 2022), and results were considered significant at *p* ≤ 0.05.

## 3. Results

### 3.1. Participant Characteristics

Nine subjects completed the lifestyle logging protocol in its entirety. Prolonged sitting ranged from 4.1 to 11.1 h per day (mean daily sitting ± *SD* = 6.77 ± 1.42). Estimated weekly physical activity ranged from 4.5 h to 12 h (mean weekly physical activity ± *SD* = 6.8 ± 2.4). Subjects’ mean daily protein and carbohydrate intake were 105.7 ± 32.8 and 190.5 ± 53.7 kcals, respectively. 

### 3.2. Descriptive Statistics

Table 1 displays means (± *SD*s) for soreness and inflammation markers. 

### 3.3. Peak Torque

**Figure 2:**
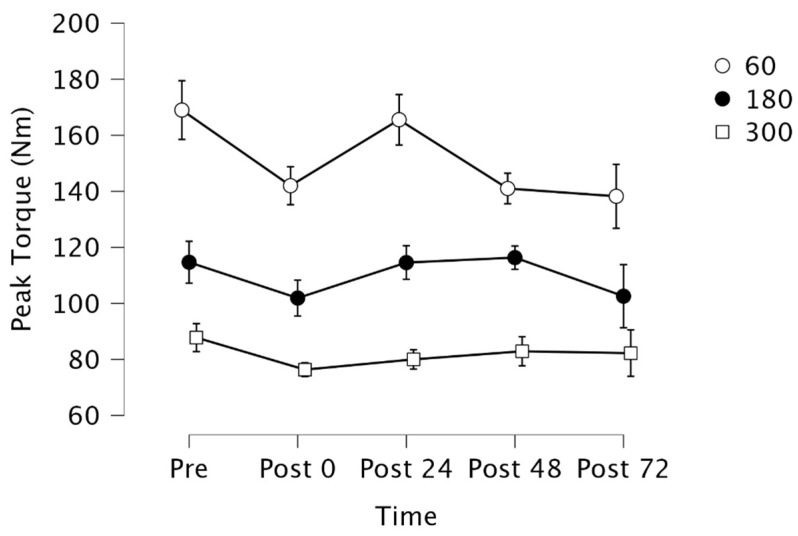
Peak torque (±*SE*) following muscle soreness-inducing protocol. Abbreviations: Pre = pre-test; 0 h = post-test; 24 h = 24 h post-test; 48 h = 48 h post-test; 72 h = 72 h post-test.

Mauchly’s test indicated that the assumption of sphericity failed for Time × Velocity ($X^{2}$ (35) = 78.609, *p* < 0.001). Therefore, degrees of freedom were corrected using Huynh-Feldt estimates of sphericity (ε = 0.694). Results indicated there was a significant two-way interaction for time × velocity (*F*[5.551, 44.405] = 2.447, *p* = 0.043) (Figure 2). Post-hoc analysis indicated a simple main effect for time at 60°·$s^{-1}$ (*p* = 0.038), but not for 180°·$s^{-1}$ or 300°·$s^{-1}$ (*p* = 0.52, *p* = 0.56, respectively). However, follow-up analysis with a Bonferroni correction demonstrated no significant differences in peak torque among the time points (*p* > 0.05).

### 3.4. Muscle Soreness and Inflammation Markers

Mean CK concentrations did not reach significance among time points (*F*[1.076, 8.608] = 4.270, *p* = 0.068). Similarly, no statistically significant differences were evident in repeated measures ANOVAs for Mb (*F*[1.088, 8.705] = 2.812, *p* = 0.128), CRP (*F*[1.269, 10.152] = 0.102, *p* = 0.814), or WBC (*F*[2.635, 21.082] = 2.071, *p* = 0.140) concentrations among time points.

Table 2 displays the correlations between lifestyle logging metrics (mean daily prolonged sitting time, weekly physical activity, and daily protein and carbohydrate intake) and indirect inflammatory and muscle damage markers (WBC, CRP, Mb, and CK). In the following days after the overload protocol, CK levels rose and peaked at 72 h, where a significant and strong positive correlation between average daily prolonged sitting time and percent increase in CK concentration from 0 h to 72 h (*r* = 0.738, *p* = 0.023) was evident. Additionally, significant and very strong positive correlations were apparent when comparing the average prolonged sitting time percent change in Mb concentration at 48 h and 72 h (*r* = 0.71, *p* = 0.033, *r* = 0.889, *p* = 0.001, respectively) and average weekly physical activity (*r* = 0.729, *p* = 0.026). Although WBC and CRP concentrations increased following the overload protocol, as evident in Table 1, neither reached significance (*p* > 0.05). As noted in Table 2, no associations were evident between either daily carbohydrate or protein intake and recovery as assessed by CK, Mb, WBC, and CRP (*p* > 0.05).

## 4. Discussion

The aims of this study were to assess changes in indirect inflammatory and muscle damage markers indicative of recovery time after an exhaustive exercise bout and to examine whether these changes are influenced by various lifestyle metrics, including average daily prolonged sitting, physical activity, and carbohydrate and protein intake. Results from this study indicated a significant association between participants who reported higher prolonged sitting time each day and heightened CK and Mb concentrations at 72 h, 48, and 72 h post-exercise, respectively. These findings suggest the recovery response to an exhaustive exercise bout may be extended in those who engage in more sitting throughout the day. This study highlights several noteworthy strengths, foremost among them being the methodology and findings presented in our study, which provide practical application for examining the impact of prolonged sitting on recovery time and underscore the need for additional studies to expand upon these initial findings. Another strength of this study was the repeated measures design, wherein each subject served as their own pre-test control. Additionally, the novel findings in our study contribute to the habitual prolonged sitting literature. To our knowledge, this is the first study to examine the recovery response from an exhaustive exercise bout compared with common lifestyle metrics in college-age individuals, a population where a significant decline in physical activity has been observed [72,73].

Research has shown that a strenuous, exhaustive exercise bout is followed by a rise in indirect inflammatory and muscle damage markers, including CRP, WBC, CK, and Mb, which may often remain elevated for several days thereafter [51,53,64]. Although both CK and Mb mean concentrations increased substantially over the measured time period in the present study, peaking at 72 h, neither marker reached statistical significance at any time point compared to baseline.

There is considerable literature on CK and Mb responses to strenuous exercise [48,84]. In an investigation using a comparable methodology to the present study, Gordon III et al. [42] reported significantly elevated Mb and Ck concentrations in the following 24 h and 24 and 48 h, respectively, following a concentric knee extension and eccentric flexion exercise protocol at 60°·$s^{-1}$. Similar findings were evident in a study by Croisier et al. [45], who demonstrated significant increases in CK and Mb at 24 h and peaking at 48 h following an isokinetic eccentric exercise of the quadriceps. Furthermore, Jenkins et al. [81] published comparable findings, with significantly elevated CK and Mb concentrations at 48 and 72 h.

Previous research indicates that eccentric exercise-induced reductions in performance and heightened muscle damage as measured through markers CK, Mb, CRP, and WBC subside within seven days [87,88,89]. This was evident in the present study, as markers and peak torque were only measured through day four, with no apparent return to baseline in the variables that displayed substantial changes. It is likely that assessing peak torque, inflammation, and muscle damage up to seven days following the overload protocol may have revealed a return to baseline.

WBC and CRP are often used to assess inflammation in the body [87,90,91], with previous research showing associations between a sedentary lifestyle and heightened WBC and CRP levels [67,92]. Healy et al. [17] demonstrated an association between prolonged sitting time and the inflammatory biomarker CRP. In the cross-sectional analysis, participants wore an accelerometer during all waking hours for seven days. CRP was detrimentally associated with prolonged sitting time and beneficially associated with breaks in prolonged sitting. Considering these findings from previous studies, it is postulated that an association between a higher average daily prolonged sitting time and elevated WBC and CRP concentrations at baseline may be evident in the findings. No such association was found in the present investigation, likely due to a variety of factors, including sample size, a lack of standardized daily prolonged sitting duration throughout the testing period, and self-reporting of sitting time.

Few studies exist examining changes in CRP following exercise, and the results that exist are mixed [42,85,93,94,95,96]. One study [93] reported an increase in CRP from 24 h to 48 h following long-distance running, while another study involving uphill and downhill running demonstrated no differences in CRP concentrations post-exercise [94]. In contrast, an investigation using a comparable methodology to the present study found no significant changes in CRP concentrations following an exhaustive exercise protocol for the quadriceps [42]. Additionally, a study by Nosaka and Clarkson [95] reported no significant differences in CRP concentrations despite heightened CK levels. Two studies with similar exercise protocols where subjects performed a bout of exercise with the elbow flexor [85,96] demonstrated no significant changes in CRP concentrations over the following 5 days compared to baseline. These findings are in support of the present study, where no significant differences in CRP concentrations were found over the assessed time period despite increased Mb and CK concentrations nearly reaching significance in addition to reduced peak torque.

Changes in WBC concentrations following exhaustive exercise also differ in the literature [85,97,98]. WBC and CK concentrations were elevated 24 h after performing an eccentric elbow extension protocol [97]. Similarly, MacIntyre et al. [98] found significantly increased WBC concentrations after an exhaustive exercise protocol of the quadriceps, although the protocol was higher in volume compared to the present study (300 repetitions vs. 100). Perhaps the overload protocol used in the present study did not induce sufficient muscle damage to significantly influence the inflammatory markers CRP and WBC. This theory is reflected in a similar study where a larger volume of repetitions produced a more pronounced inflammatory response, resulting in significantly elevated WBC and CRP concentrations [85]. Furthermore, the large differences in results in the current study between participants produced large standard errors, likely impacting the findings. As highlighted in a study by Nosaka and Clarkson [95], the magnitude of muscle damage and inflammation can vary significantly across participants. Future studies should aim to assess a larger group of participants to minimize large standard errors.

In contrast to a similar overload protocol using the elbow flexor conducted by Newton et al. [48], where a significant decrease in peak torque from pre- to post-overload protocol was evident at a velocity of 90°·$s^{-1}$, the current study found no differences in peak torque across time points or velocities, excluding a simple main effect at 60°·$s^{-1}.$ The lack of other significant declines in peak torque may indicate that the intended muscle damage extent may not have been reached, therefore perhaps not affecting the inflammation and muscle damage marker outcomes. The reason for the lack of change in peak torque could possibly be in part due to participants not exerting maximal effort through all 10 sets of eccentric contractions during the overload protocol. Not only could this affect the level of damage to the quadriceps and, thus, impact peak torque following the overload protocol, but the inflammatory and muscle damage markers as well.

No correlations were evident between daily carbohydrate or protein intake and recovery time, as assessed by CK, Mb, CRP, and WBC concentrations at baseline and the three days following the exhaustive exercise bout. Little is known about macronutrient intake differences during recovery from exhaustive exercise in terms of muscle damage and inflammatory responses. In a cross-over design study, participants completed eccentric contractions of the elbow flexors and extensors following either a high- or low-carbohydrate diet. CK levels increased from pre- to 24 h and 120 h but did not reach significance, although perceived soreness and interleukin-1β were higher at all time points post-exercise in the high carbohydrate group. The author’s conclusion was that inflammation was greater with a high-carbohydrate diet compared to a low-carbohydrate diet [85]. Additional studies have found similar findings, with carbohydrate status appearing to have little to no impact on recovery time as measured by muscle damage markers [98,99,100]. In contrast, Cockburn et al. [43] used a protocol similar to the present study. Participants were given one of four liquids to consume following the eccentric-exercise bout [43]. The results suggested at 48 h following the eccentric exercise bout that milk and milk-based protein and carbohydrate supplementation resulted in attenuation of decreases in isokinetic muscle performance and increases in CK and Mb [43].

To our knowledge, no studies to date have investigated the association between average daily protein intake and recovery after performing an exhaustive exercise overload protocol on the quadriceps. The existing research on protein intake and recovery from an exhaustive protocol differs significantly in methodology from the present study and predominantly examines the impact of additional supplementation in contrast to average daily intake [101]. Additional well-controlled studies are needed to explore the possible association between inflammation and muscle damage following an exhaustive exercise protocol and daily protein intake.

One notable limitation of this study was the relatively small sample size, which contributed to potential variability in the data and resulted in larger standard deviations and outliers. It is crucial to underscore that the inclusion of only nine subjects in our sample renders it insufficient to provide definitive conclusions. Therefore, additional well-controlled studies with significantly larger numbers of participants are needed to support the study findings and validate the robustness of the methodology. Another limitation was the use of self-reporting for dietary [102], physical activity [78,103], and prolonged sitting logs [104]. Future studies could use an accelerometer to track daily prolonged sitting time and physical activity more accurately and improve dietary intake variability by requiring specific macronutrient guidelines or providing identical meals to all participants. Additionally, we did not assess hydration status, and thus, future studies should include this measure to assess the potential impact hydration may contribute. Lastly, the correlation analysis of biomarkers among the different time points based on percentage of change must be analyzed with caution, given that percent change correlations might be prone to producing misleading results [105]. A potential research direction could follow a similar protocol in women or compare the impact of prolonged sitting on performance and recovery in men and women. Moreover, a prospective avenue for future research entails an exploration of the discernible variations in response among resistance-trained individuals or athletes in contrast to their recreationally trained or untrained counterparts. An additional proposed research direction involves adopting a comparable study design with a focus on aerobic exercise to analyze research outcomes concerning the impact of resistance exercise compared to aerobic exercise on time to recovery, as assessed through systemic inflammation and muscle damage markers.

## 5. Conclusions

This study suggests greater daily prolonged sitting time may be associated with extended time to recovery as assessed by the heightened muscle damage markers CK and Mb following a strenuous exercise bout. Average weekly physical activity, as well as daily carbohydrate and protein intake, had no beneficial effects on reducing Mb, CK, CRP, or WBC. Although the unilateral isokinetic muscle actions of the quadriceps caused measurable inflammation and muscle damage, it was not enough to reach statistical significance. In contrast, previous research [42,43,81] has indicated significantly decreased inflammatory and muscle damage markers upon completing a comparable overload exercise protocol, and thus, the results should be interpreted with caution. Consequently, it is still unknown whether an association between recovery time and daily average macronutrients or daily activity level, following an exhaustive exercise bout, exists. In summary, greater daily prolonged sitting was associated with an extended period of muscular recovery following an exhaustive bout of resistance exercise. Therefore, minimizing extended periods of sitting throughout the day may be beneficial for reducing recovery time after a strenuous exercise protocol.

## Figures and Tables

**Table 1:** Blood markers following muscle soreness inducing protocol (*M ± SD*).

Variable	Pre	0 h	24 h	48 h	72 h
WBC (K/μL)	6.41 ± 2.97	7.31 ± 2.49	6.73 ± 1.58	6.28 ± 1.71	6.21 ± 1.29
CRP (mg/L)	2.08 ± 3.09	2.08 ± 3.18	2.2 ± 2.89	2.1 ± 2.62	1.79 ± 2.58
CK (U/L)	366.00 ± 464.48	365.00 ± 416.44	414.56 ± 286.98	622.33 ± 349.77	2112.33 ± 2347.3
Mb (mcg/L)	45.11 ± 22.52	57.22 ± 23.81	47.89 ± 21.99	247.22 ± 286.04	422.89 ± 670.45

Abbreviations: CK = creatine kinase; CRP = C-reactive protein; Mb = myoglobin; WBC = white blood cells.

**Table 2:** Associations between lifestyle logging and recovery as measured by soreness and inflammatory blood markers.

Variable	0–24 h		0–48 h		0–72 h	
	*r*	*p*	*r*	*p*	*r*	*p*
Sitting Time						
WBC (K/μL)	0.558	0.118	0.551	0.124	0.465	0.207
CRP (mg/L)	0.010	0.979	0.001	0.997	−0.024	0.951
CK (U/L)	0.232	0.548	0.496	0.175	0.738 *	0.023 *
Mb (mcg/L)	0.280	0.466	0.71 *	0.033 *	0.889 **	0.001 **
Physical Activity						
WBC (K/μL)	0.517	0.154	0.399	0.287	0.310	0.417
CRP (mg/L)	0.176	0.650	0.157	0.687	0.105	0.789
CK (U/L)	−0.057	0.885	0.081	0.835	0.469	0.203
Mb (mcg/L)	−0.157	0.688	0.388	0.303	0.729 *	0.026 *
Protein Intake						
WBC (K/μL)	0.218	0.573	0.211	0.585	0.562	0.116
CRP (mg/L)	0.299	0.435	0.179	0.645	0.217	0.575
CK (U/L)	−0.173	0.656	0.251	0.514	0.042	0.915
Mb (mcg/L)	0.278	0.468	0.112	0.775	−0.096	0.807
Carbohydrate Intake						
WBC (K/μL)	0.18	0.642	0.642	0.063	0.177	0.649
CRP (mg/L)	0.127	0.745	0.138	0.724	0.120	0.759
CK (U/L)	0.170	0.661	0.513	0.157	0.551	0.124
Mb (mcg/L)	−0.078	0.842	0.479	0.192	0.208	0.592

* Denotes a significant correlation between different time points. * *p* < 0.05; ** *p* < 0.01. Abbreviations: CK = creatine kinase; CRP = C-reactive protein; Mb = myoglobin; WBC = white blood cells. All correlations are measured as a percent difference between 0 h and 24 h, 0 h and 48 h, and 0 h and 72 h.

## Data Availability

Data will be made available upon reasonable request.
